# Supplementary material for: Prostaglandin EP2 receptor downstream of Notch signaling inhibits differentiation of human skeletal muscle progenitors in differentiation conditions
Source: Commun Biol. 2020 Apr 20;3:182. doi: 10.1038/s42003-020-0904-6 (PMC7171165; doi:10.1038/s42003-020-0904-6)
Supplement: Supplementary file 1 — Supplementary Information [file 42003_2020_904_MOESM1_ESM.pdf]

Supplementary Figures & Tables

Sakai-Takemura *et al.*
Prostaglandin EP2 receptor downstream of NOTCH signaling inhibits differentiation of human skeletal muscle progenitors in differentiation conditions

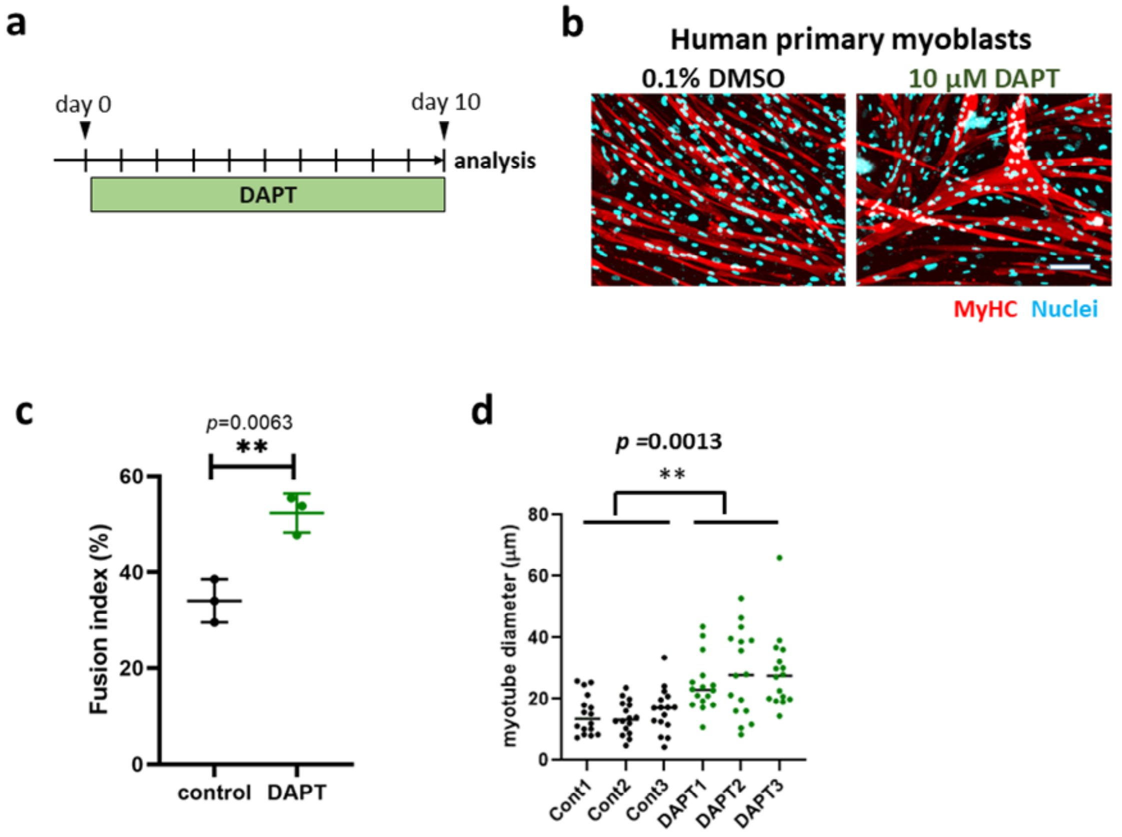

Supplementary Fig. 1 (related to Fig. 1)
DAPT treatment improves fusion of human primary myoblasts.

a. Experimental design.

b. Representative photos of myotubes formed by human primary myoblasts after 1-week treatment with DAPT (10 μM). Cells were stained with MF20 (skeletal muscle myosin, red) and DAPI (nuclei, blue). Scale bar, 100 μm.

c. Fusion index of human primary myoblasts. Data are shown as mean ± S.D. (n=3 samples/ group) and compared by unpaired two-tailed Student's *t*-test.

d. Distribution of diameter of myotubes formed by human primary myoblasts. n=3 samples/group. Average of the samples was compared by unpaired two-tailed Student's *t*-test.

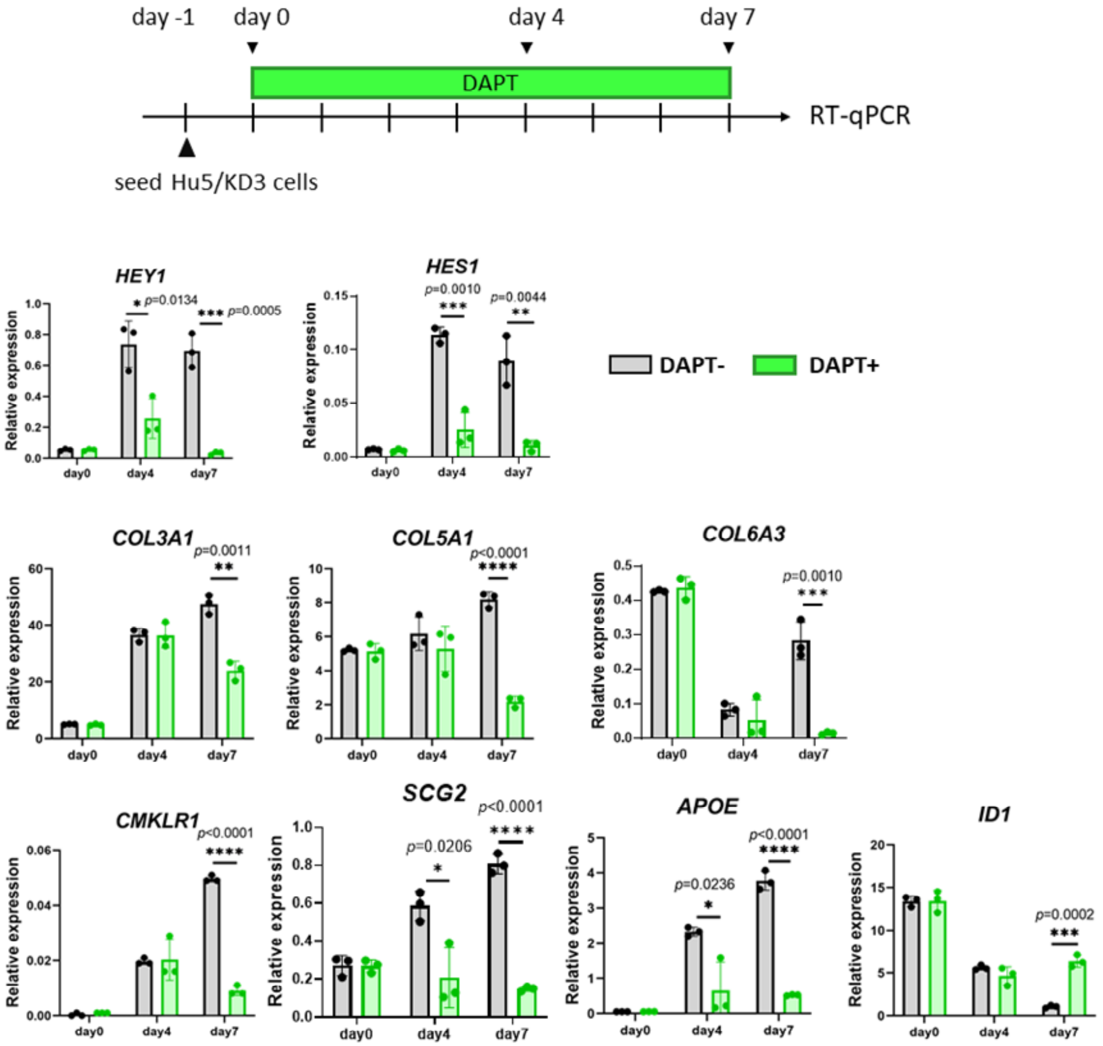

Supplementary Fig. 2 (related to Fig. 3)

Gene expression in DAPT-treated and non-treated Hu5/KD3 human muscle progenitors.

a. Experimental design and qPCR analysis of *HEY1, HES1, HEYL, COL3A1, COL5A1, COL6A3, SCG2, APOE, CMKLR1*, and *ID1* in Hu5/KD3 muscle progenitors with or without DAPT treatment. Cells were seeded at $5x10^4$/well of 24-well plates and treated with DAPT for 7 days. Total RNA was extracted from the cells at day 0, day 4 and day 7. Data are shown as mean ± S.D. (n=3 samples/group). DAPT-treated groups were compared with controls by unpaired two-tailed Student's *t*-test.

a

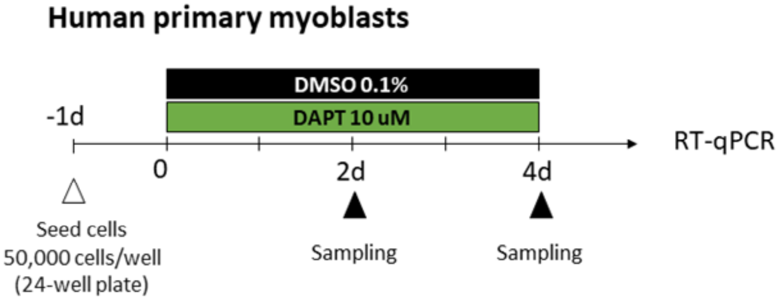

b

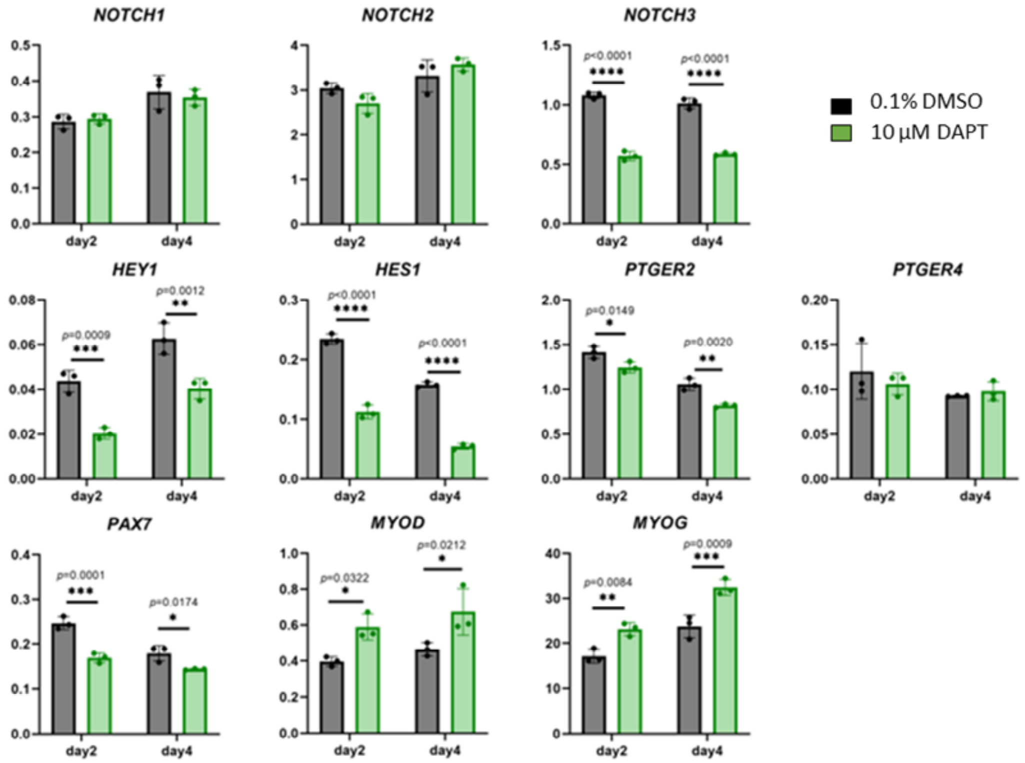

Supplementary Fig. 3 (related to Fig. 3)

Analysis of gene expression in DAPT-treated and non-treated human primary myoblasts. **a**, Experimental design. **b,** RT-qPCR analysis of NOTCH1, NOTCH2, NOTCH3, *HEY1, HES1, PTGER2, PTGER4, PAX7, MYOD,* and *MYOG* in human primary myoblasts with or without DAPT treatment. Cells were seeded at $5x10^4$/well of 24-well plates and treated with DAPT for 4 days. Total RNA was extracted from the cells at days 2 and 4. Data are shown as means ± S.D. (n=3 samples/group). DAPT-treated groups were compared with controls by unpaired two-tailed Student's t-test.

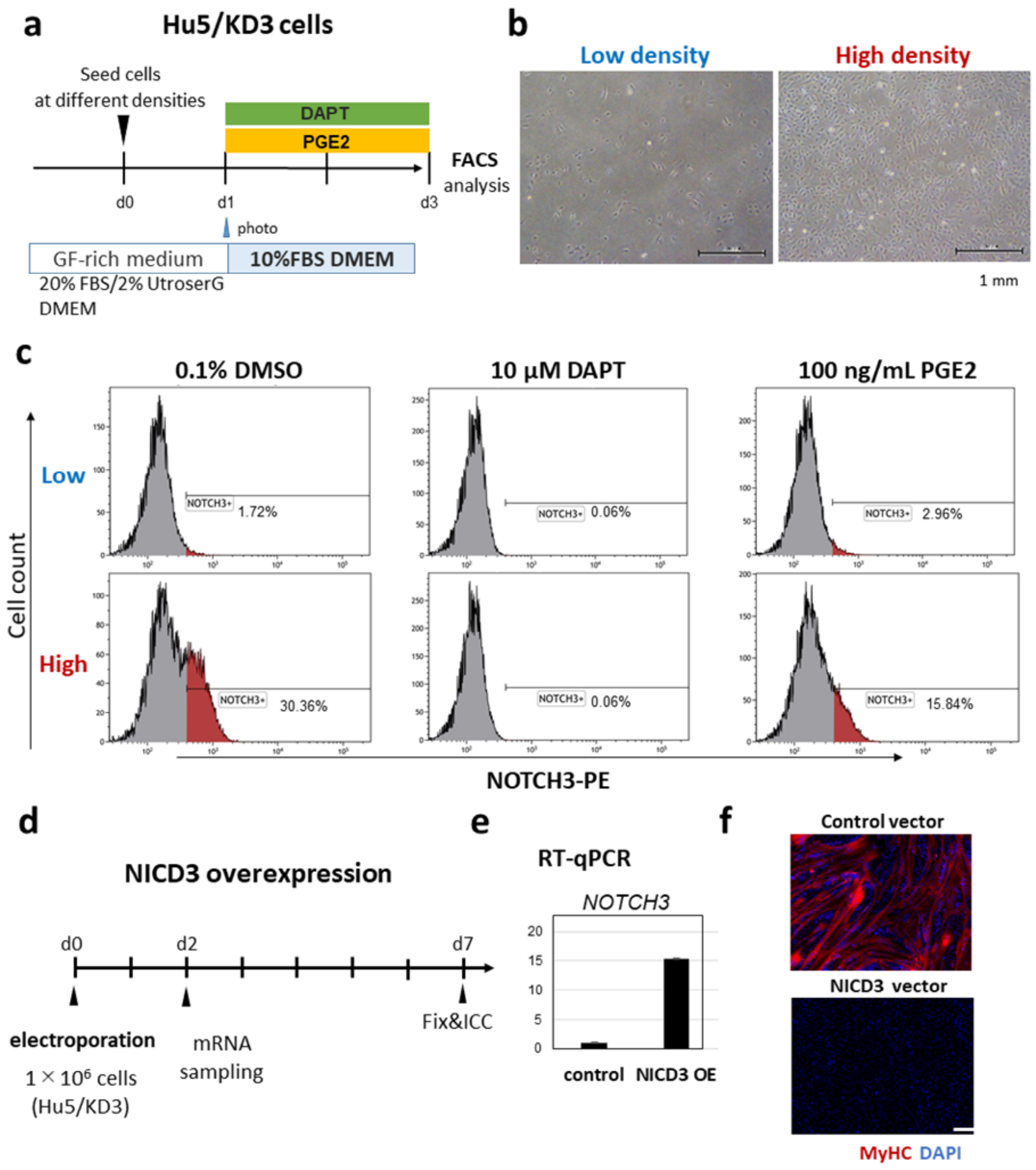

Supplementary Fig. 4 (related to Fig. 4)

***NOTCH3* expression is induced in human muscle progenitors in high cell density culture**

a. Experimental design. Hu5/KD3 cells were plated onto collagen I-dishes at two different cell densities: 5000 cells/cm^2 (low density) and 25000 cells/cm^2 (high density) (d0). Cells were cultured in the presence of DAPT (10 µM) or PGE2 (100 ng/ml) for 2 days (d1-d3).

b. Phase contrast of Hu5/KD3 cells 24 h after plating at different cell densities. Bar: 1

mm.

c. FACS for NOTCH3 expression on Hu5/KD3 plated at different cell densities. When plated at high cell density, a fraction of Hu5/kd3 cells started to express NOTCH3. DAPT treatment abolished NOTCH3 induction. PGE2 down-regulated NOTCH3 expression.

d. Experimental design of NICD3 overexpression. Ten µg of plasmids were introduced into Hu5/KD3 cells ($1x10^6$) using electroporation.

e. RT-qPCR for *NOTCH3* (n=1). At day2, cells were harvested and RNA was extracted.

f. Representative immunostaining of Hu5/KD3-myotubes transfected with control vector (control) or NICD3 expression vector. NICD3 overexpression completely inhibited myotube formation by Hu5/KD3 cells. Scale bar, 200 µm.

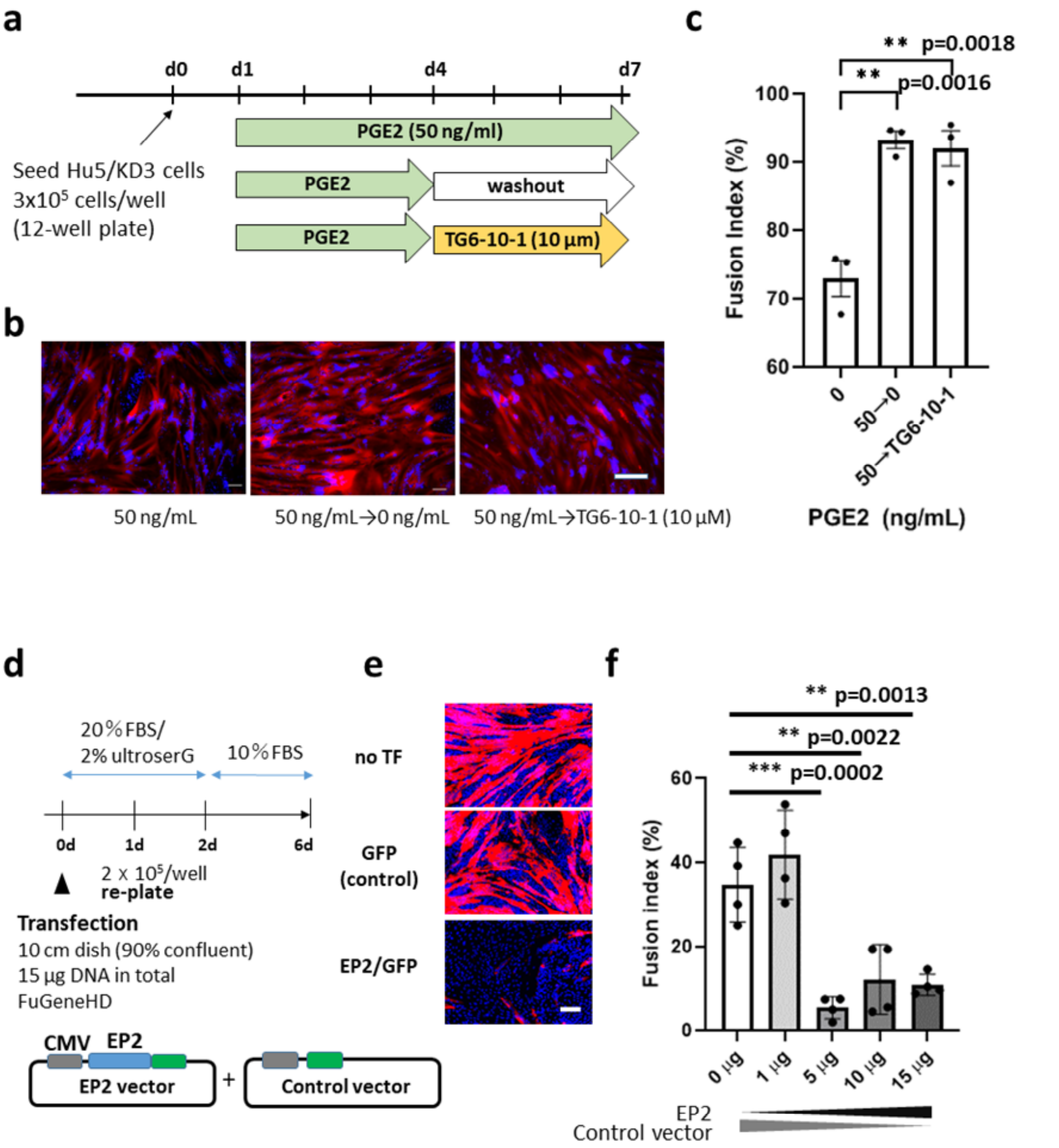

Supplementary Fig. 5 (related to Fig. 5&6)

Reversibility of effects of prostaglandin E2 (PGE2) on myotube formation of a human myoblast cell line, Hu5/KD3 cells

a. Experimental design. Hu5/KD3 cells were seeded onto collagen type I-coated 12-well plates ($3x10^5$ cells/well) and divided into three groups. All groups were cultured in 10% FBS/DMEM medium with PGE2 (50 ng/ml) for 3 days. The first group was cultured continuously with PGE2 (50 ng/ml) until day 7. In the second group, PGE2 was removed at day 4. In the third group, PGE2 was changed to TG6-10-1 (10 µM) at

day 4. All groups were fixed at day 7. n=3/group.

- **b.** Representative myosin staining of differentiating Hu5/KD3 cells in the three groups.
- **c.** Fusion index of Hu5/KD3 cells after treatment. Data are shown as mean ± S.D. (n=3 samples). Comparison with controls was performed by one-way ANOVA followed by Dunnett's multiple comparisons test.
- **d.** Experimental design. Hu5/KD3 cells (90% confluent in 100 mm collagen-coated dishes) were transfected with CMV-GFP and CMV-EP2-GFP (15 μg in total) and cultured in differentiation medium.
- **e.** Representative myotube formation by Hu5/KD3 cells without transfection, Hu5/KD3 cells transfected with CMV-GFP (CMV-GFP: 15 μg), or Hu5/KD3 cells transfected with CMV-EP2-GFP (15 μg).
- **f.** Fusion index. The doses of CMV-EP2-GFP are shown below the graph. n=4 samples/group. Comparison with controls (EP2 vector: 0 μg) was performed by one-way ANOVA followed by Dunnett's multiple comparisons test. In **b** and **e**, cells fixed with 4% PFA were stained with MF20 (muscle myosin heavy chain, red) and DAPI (nuclei, blue). Scale bar, 100 μm.

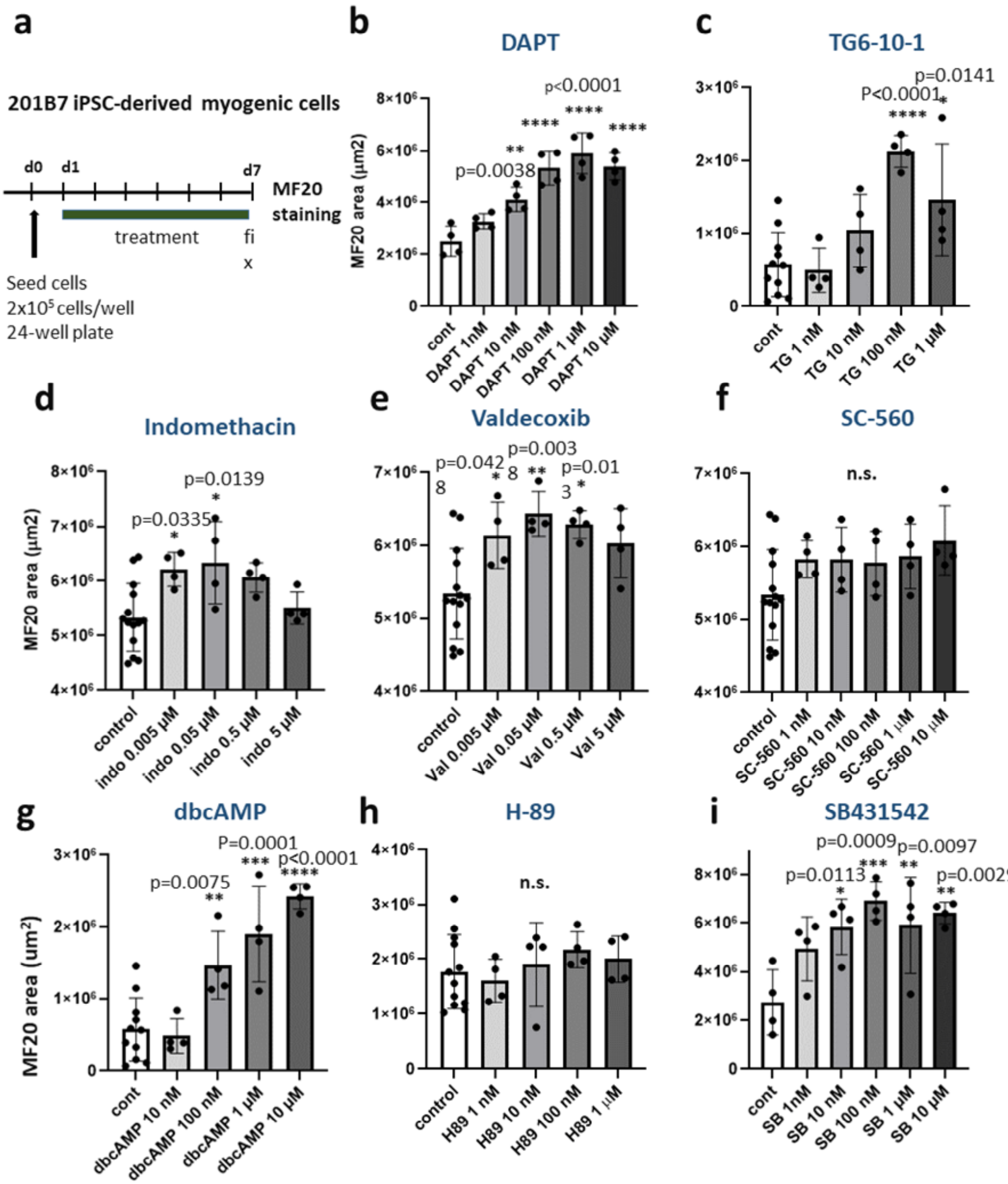

Supplementary Figure 6 (related to Fig. 8)

Effects of inhibition or activation of signaling molecules upstream and downstream of EP2 on differentiation of hiPSC-derived muscle progenitors.

a. Experimental design.

b-i, 201B7 hiPSC-derived muscle progenitors seeded at 2x10^5 cells/well of collagen-coated 24-well plates were treated with DAPT(**b**). TG6-10-1(**c**), Indomethacin(**d**), Valdecoxib (**e**), SC-560 (**f**), dbcAMP (**g**), H-89(**h**), or SB431542 (**i**) at indicated concentrations. n≧4

samples/ group. Four views were recorded per sample. MF20-positive area was measured. One-way ANOVA followed by Dunnett's multiple comparisons test (versus control). ns, not significant.

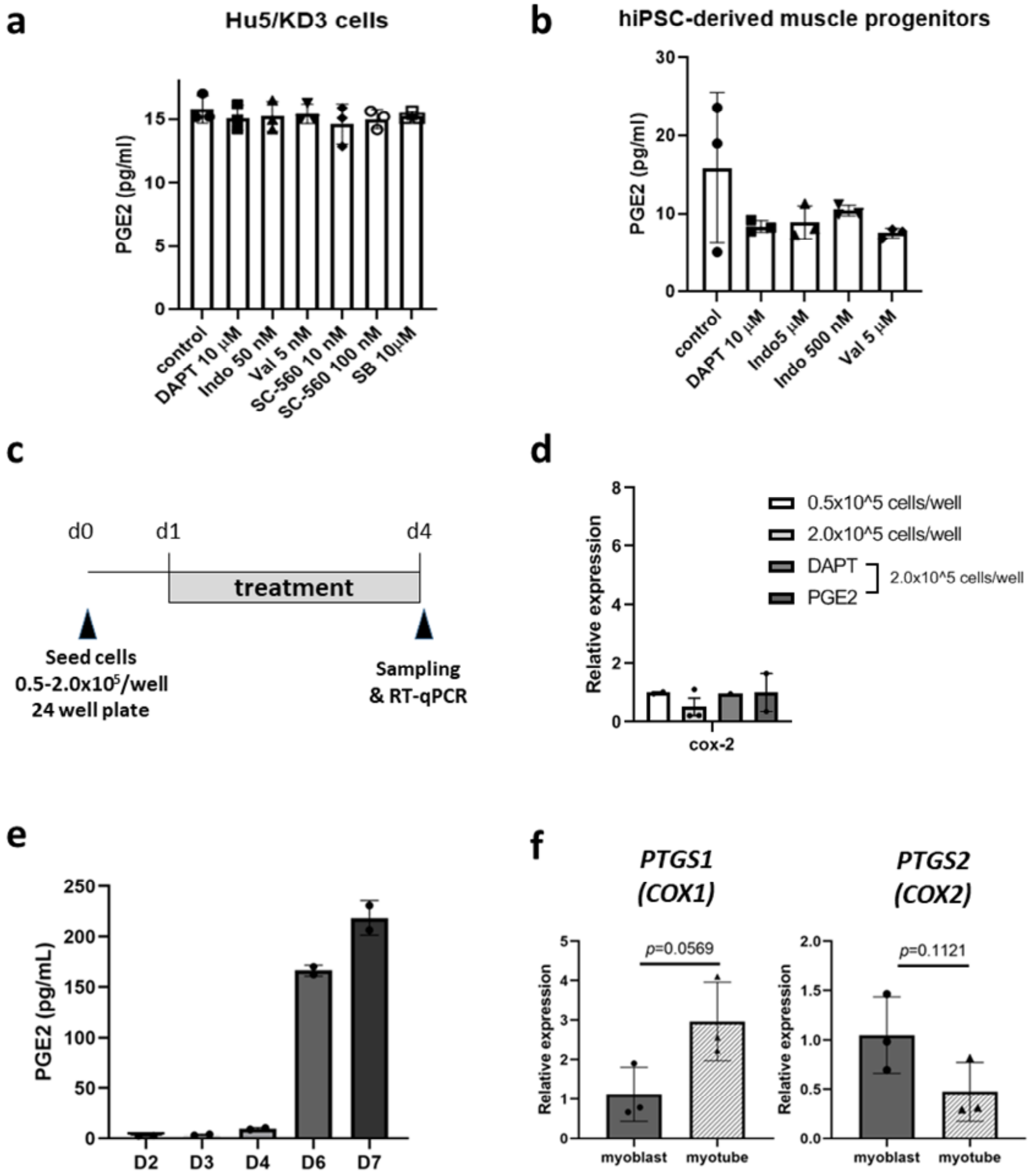

Supplementary Fig. 7

Concentration of PGE2 (culture medium) and *COX2* mRNA levels in DAPT- or COX-inhibitor-treated cells.

a., b. PEG2 concentration in the culture medium of Hu5/KD3 (a) or hiPSC-derived muscle progenitors (b) cultured in the presence of DAPT (10 μM), indomethacin (500 nM, 5 μM), valdecoxib (5 μM), SC-760 (10 nM, 100 nM) or SB431542 (10 μM) for 48 h (**a**) or 24 h (**b**). The concentration was measured using a PGE2 Elisa kit (Cayman). n=3 samples/group.

One-way ANOVA with Dunnett's multiple comparisons test.

c., d. RT-qPCR for *COX2 (PTGS2)* mRNA in proliferating (plated at $0.5x10^5$ cells/well, 6-well plate) and differentiating ($2.0x10^5$ cells/well) Hu5/KD3 cells treated with DAPT (10 μM) or PGE2 (50 ng/ml).

e, PGE2 levels during muscle differentiation. At day 4 (D4) myotubes started to form and at day6 (D6) or day 7 (D7), robust formation of multinucleated myotubes were observed. PGE2 levels increased with differentiation. n=2 samples/group.

f, *COX1 (PTGS1)* and *COX2* mRNA levels in myoblasts and myotubes. Although there is statistically no significant difference, *COX2* expression was higher in myoblasts than in myotubes. In contrast, *COX1* expression was higher in myotubes than in myoblasts. n=3 samples/group. Unpaired two-tailed Student's t-test.

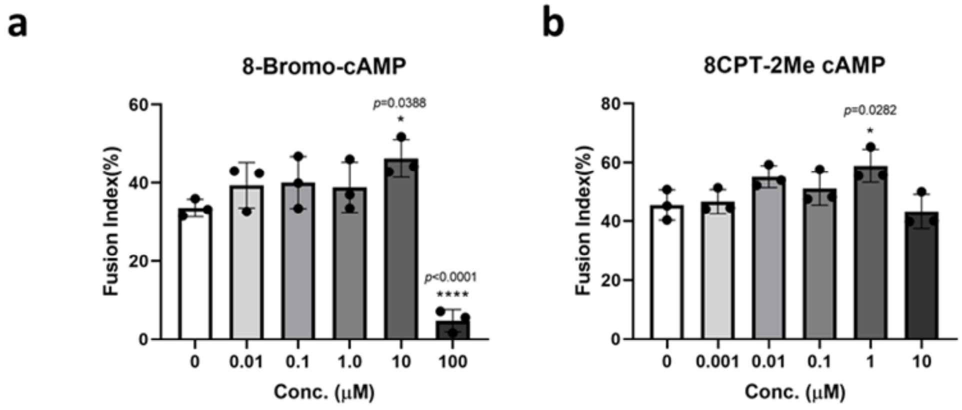

Supplementary Fig. 8 (related to Figure 8)
Effects of cAMP derivatives on differentiation of Hu5/KD3 cells

Effects of 8-bromo-cAMP (selective activator of protein kinase A) on differentiation of Hu5/KD3 cells. High concentration of 8-bromo-cAMP (100 μM) showed toxic effects on the cells. n=3 samples/group.

a. Effects of 8-CPT-2Me-cAMP (selective Epac activator) on differentiation of Hu5/KD3 cells. n=3 samples/group. In **a** and **b**, one-way ANOVA with Dunnett's multiple comparisons test was performed (versus control).

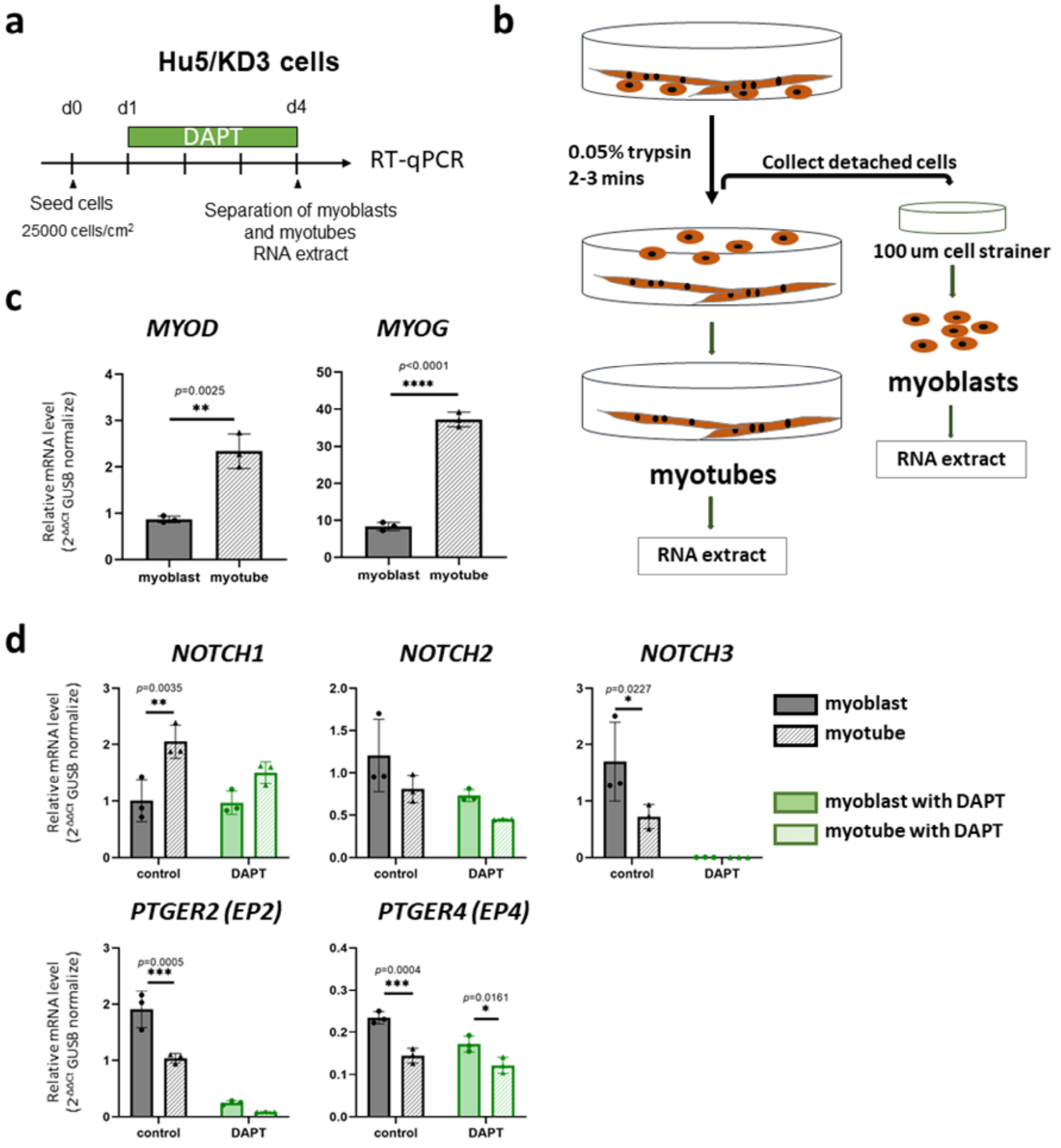

Supplementary Fig. 9

Expression levels of *NOTCH2, NOTCH3, PTGER2* and *PTGER4* are higher in myoblasts than in myotubes

a. Experimental design. Hu5/KD3 cells (p31) were cultured for 3 d. Then formed multinuclear myotubes were separated from mononuclear cells as shown in **b** for RT-qPCR analysis.

b. Differentiating myogenic cells were treated with 0.05% trypsin for 2 mins at RT. Detached mononuclear cells were collected and passed through 100-µm cell strainers

(myoblasts). Myotubes that remained attached to dishes were collected using a cell scraper (myotubes).

c. Relative levels of *MYOD* and *MYOGENIN* mRNA in “myoblasts” and “myotubes”.

d. Relative levels of *NOTCH1, NOTCH2, NOTCH3, PTGER2 (EP2)*, and *PTGER4(EP4)* mRNA in myoblasts and myotubes with or without DAPT treatment. In **c** and **d**, 3 samples were examined per group. Unpaired two-tailed Student's t-test.

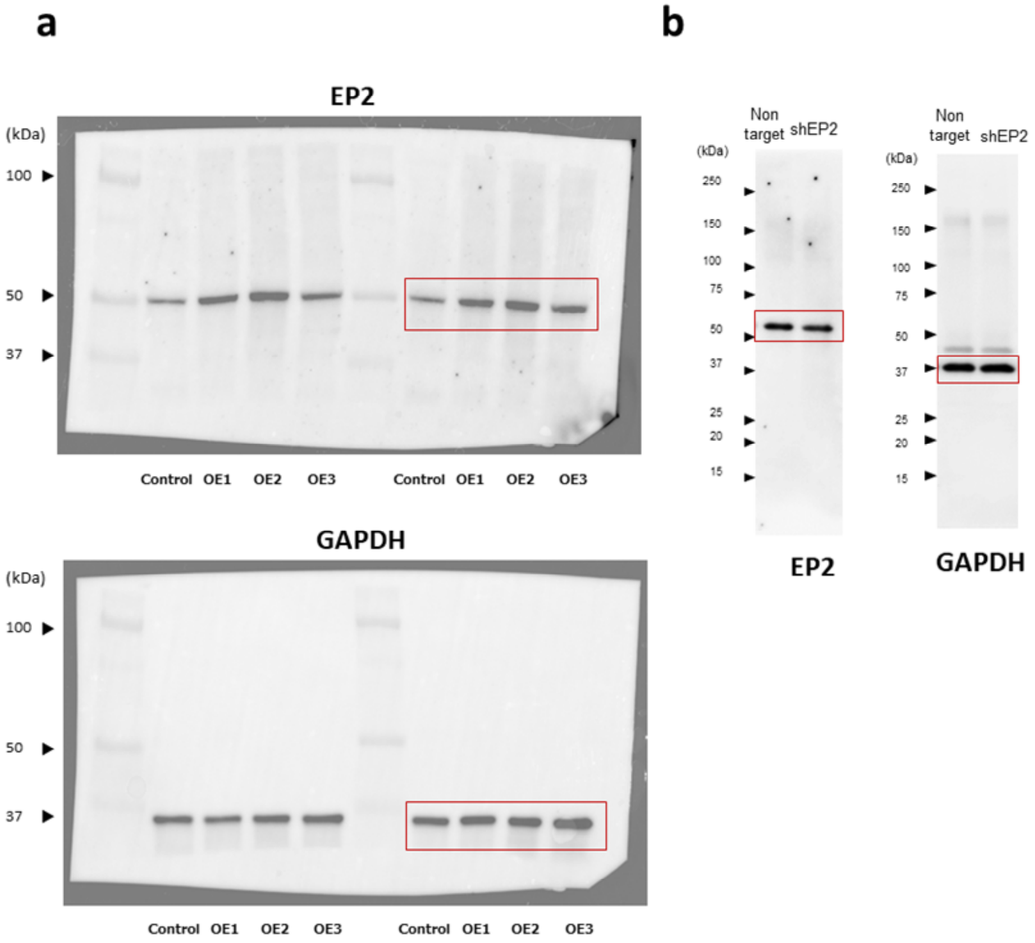

Supplementary Fig. 10

Whole images of Western blots in Figure 6

a. EP2 overexpression experiments.

b. EP2 knockdown by shRNA vectors. EP2 and GAPDH (loading control) were detected as a single band of approximately 50 kDa and 37 kDa, respectively.

Supplementary Table 1

Primers for RT-qPCR

GENE name	Forward (5'-3')	Reverse (5'-3')	bp
NOTCH1	GGTGAACTGCTCTGAGGAGATC	GGATTGCAGTCGTCCACGTTGA	150
NOTCH2	GGGACCCTGTCATACCCTCT	GAGCCATGCTTACGCTTTCG	155
NOTCH3	CAACCTGGCAGGGAGTTTCA	TCAGGCACTCATCCACATCG	197
HEY1	GAAGCGCCGACGAGACCGAATCAA	CAGGGCGTGCGCGTCAAAATAACC	193
HES1	GGAAATGACAGTGAAGCACCTCC	GAAGCGGGTCACCTCGTTCATG	130
HEYL	GAGAGTGCGGACGAGAATGG	GATCCTTGCTCCATTACCTGC	197
PTGER2	GACCACCTCATTCTCCTGGCTA	AACCTAAGAGCTTGGAGGTCCC	128
PTGER4	CCGCTCGTGGTGCGAGTATT	GAGATGAAGGAGCGAGAGTGG	287
COL3A1	CTTCTCTCCAGCCGAGCTTC	TGTGTTTCGTGCAACCATCC	187
COL5A1	TTCAAGCGTGGGAAAACTGCT	GCCTTTCTTGGTAGCACAGC	265
COL6A3	TATCCACCTCCAGCAGTTGAG	CTCTCACTTTACTGGGGCCG	151
APOE	GGACAGGGGGAGCCCTATAA	GTGATTGGCCAGTCGGCTC	105
CMKLR1	AGGGACTGATTGGCTGAGGA	ATCCTCCATTCTCATTCACCGT	70
UNC5B	TGTGCATGCAAATGCTGGAG	TGTCTGTGTCGAAGTCACGG	141
SCG2	AGGGATGGAGAGTGCAGCAAATCA	AGCTTTGGGAAGCTGGTTCGATCT	124
ID1	ATTACGTGCTCTGTGGGTCTCC	TAGTAGGTGTGCAGAGAGGAGC	153
GAPDH	GCACCGTCAAGGCTGAGAAC	TGGTGAAGACGCCAGTGGA	138
GUSB	AACGATTGCAGGTTTCAC	CTCTCGTCGGTGACTGTTCA	171
MYOD	GGGGCTAGGTTCAGCTTTCT	GCTCTGGCAAAGCAACTCTT	233
MYOGENIN	QIAGEN primers cat#: QT00001722* Sequences are not disclosed		
PAX7	GGCGACTCCGGATGTAGAGA	AGCACGCGGCTAATCGAAC	153
PTGS1 (COX1)	GAGTACTGGAAGCCGAGCAC	AGGGACAGGTCTTGGTGTTG	103
PTGS2 (COX2)	GCAGTTGTTCCAGACAAGCA	GAAAGGTGTCAGGCAGAAGG	209
