# Supplementary material for: Prostaglandin EP2 receptor downstream of Notch signaling inhibits differentiation of human skeletal muscle progenitors in differentiation conditions
Source: Commun Biol. 2020 Apr 20;3:182. doi: 10.1038/s42003-020-0904-6 (PMC7171165; doi:10.1038/s42003-020-0904-6)
Supplement: Supplementary file 3 — Description of Additional Supplementary Files [file 42003_2020_904_MOESM3_ESM.pdf]

Descriptions of Additional Supplementary Files

Raw values of the experimental data presented in the article are shown in Supplementary Data (50 sheets) with statistical parameters.

Data for Figure 1d
Data for Figure 1e
Data for Figure 1h
Data for Figure 1i
Data for Figure 2a-c
Data for Figure 2d-f
Data for Table 1
Data for Figure 3c
Data for Figure 4c (Fusion index)
Data for Figure 4c (Myogenin-positive cells)
Data for Figure 4c (cell number)
Data for Figure 4d
Data for Figure 5c
Data for Figure 5e
Data for Figure 5f
Data for Figure 5g
Data for Figure 6a-d
Data for Figure 6e-h
Data for Figure 7c
Data for Figure 7d
Data for Figure 8b
Data for Figure 8c
Data for Figure 8d
Data for Figure 8e
Data for Figure 8f
Data for Figure 8g
Data for Figure 8h
Data for Figure 8j
Data for Figure 8l
Data for Supplementary Figure 1c
Data for Supplementary Figure 1d
Data for Supplementary Figure 2
Data for Supplementary Figure 3
Data for Supplementary Figure 5a-c
Data for Supplementary Figure 5d-f
Data for Supplementary Figure 6b
Data for Supplementary Figure 6c
Data for Supplementary Figure 6d
Data for Supplementary Figure 6e
Data for Supplementary Figure 6f
Data for Supplementary Figure 6g
Data for Supplementary Figure 6h
Data for Supplementary Figure 6i

Data for Supplementary Figure 7a
Data for Supplementary Figure 7b
Data for Supplementary Figure 8a
Data for Supplementary Figure 8b
Data for Supplementary Figure 9c
Data for FACS gating
Data related to Figure 5
