# Supplementary material for: Prostaglandin EP2 receptor downstream of Notch signaling inhibits differentiation of human skeletal muscle progenitors in differentiation conditions
Source: Commun Biol. 2020 Apr 20;3:182. doi: 10.1038/s42003-020-0904-6 (PMC7171165; doi:10.1038/s42003-020-0904-6)
Supplement: Supplementary file 4 — Reporting Summary [file 42003_2020_904_MOESM4_ESM.pdf]

nature research

Corresponding author(s): Yuko Miyagoe-Suzuki

Last updated by author(s): 2020/2/24

Reporting Summary

Nature Research wishes to improve the reproducibility of the work that we publish. This form provides structure for consistency and transparency in reporting. For further information on Nature Research policies, see Authors & Referees and the Editorial Policy Checklist.

Statistics

For all statistical analyses, confirm that the following items are present in the figure legend, table legend, main text, or Methods section.

n/a	Confirmed	
☐	☒	The exact sample size (*n*) for each experimental group/condition, given as a discrete number and unit of measurement
☐	☒	A statement on whether measurements were taken from distinct samples or whether the same sample was measured repeatedly
☐	☒	The statistical test(s) used AND whether they are one- or two-sided<br>*Only common tests should be described solely by name; describe more complex techniques in the Methods section.*
☒	☐	A description of all covariates tested
☒	☐	A description of any assumptions or corrections, such as tests of normality and adjustment for multiple comparisons
☐	☒	A full description of the statistical parameters including central tendency (e.g. means) or other basic estimates (e.g. regression coefficient) AND variation (e.g. standard deviation) or associated estimates of uncertainty (e.g. confidence intervals)
☒	☐	For null hypothesis testing, the test statistic (e.g. *F*, *t*, *r*) with confidence intervals, effect sizes, degrees of freedom and *P* value noted<br>*Give P values as exact values whenever suitable.*
☒	☐	For Bayesian analysis, information on the choice of priors and Markov chain Monte Carlo settings
☒	☐	For hierarchical and complex designs, identification of the appropriate level for tests and full reporting of outcomes
☐	☒	Estimates of effect sizes (e.g. Cohen's *d*, Pearson's *r*), indicating how they were calculated

Our web collection on statistics for biologists contains articles on many of the points above.

Software and code

Policy information about availability of computer code

Data collection	All-in-one Keyence microscope (BZ-X810), FACSAria Fusion (BD), BioRad CFX connect qPCR system, BioRad ChemiDoc MP imaging system, Bio Tek plate reader
Data analysis	Image-J (NIH), Hybrid Cell Count (Keyence), GraphPad Prism8, FlowJo (BD), BioRad CFX, Microsoft Excel.

For manuscripts utilizing custom algorithms or software that are central to the research but not yet described in published literature, software must be made available to editors/reviewers. We strongly encourage code deposition in a community repository (e.g. GitHub). See the Nature Research guidelines for submitting code & software for further information.

Data

Policy information about availability of data

All manuscripts must include a data availability statement. This statement should provide the following information, where applicable:

- Accession codes, unique identifiers, or web links for publicly available datasets
- A list of figures that have associated raw data
- A description of any restrictions on data availability

All the data of this study are shown in the main text and supplementary information files. The source data underlying the main figures are presented in Supplementary Data. Any additional source data or material used in this study can be obtained from the corresponding author upon reasonable request.

Field-specific reporting

Please select the one below that is the best fit for your research. If you are not sure, read the appropriate sections before making your selection.

☒ Life sciences ☐ Behavioural & social sciences ☐ Ecological, evolutionary & environmental sciences

For a reference copy of the document with all sections, see nature.com/documents/nr-reporting-summary-flat.pdf

Life sciences study design

All studies must disclose on these points even when the disclosure is negative.

Sample size	We decided the sample size which we think is enough to show biological importance.
Data exclusions	We did not exclude any data from the analysis in this manuscript.
Replication	We repeated the experiments to confirm the reproducibility. Samples were prepared >3
Randomization	We did not randomize the samples.
Blinding	The investigators were not blinded to group allocation.

Reporting for specific materials, systems and methods

We require information from authors about some types of materials, experimental systems and methods used in many studies. Here, indicate whether each material, system or method listed is relevant to your study. If you are not sure if a list item applies to your research, read the appropriate section before selecting a response.

Materials & experimental systems

n/a	Involved in the study	
☐	☒	Antibodies
☐	☒	Eukaryotic cell lines
☒	☐	Palaeontology
☐	☒	Animals and other organisms
☒	☐	Human research participants
☒	☐	Clinical data

Methods

n/a	Involved in the study	
☒	☐	ChIP-seq
☐	☒	Flow cytometry
☒	☐	MRI-based neuroimaging

Antibodies

Antibodies used	We described all antibody names, clone names, fluorochromes, dilution, and suppliers in Methods. All antibodies were purchased from companies.
Validation	All antibodies we used have already been used in several literatures. We carefully checked datasheets provided from companies, especially whether the Ab is human protein specific or not. We included positive control (wild-type TA muscle) and negative control (dystrophin- deficient mdx TA muscle or TA muscles without transplantation).

Eukaryotic cell lines

Policy information about cell lines

Cell line source(s)	We described the sources of the cell lines: Hu5/KD3 was established by Prof. Hashimoto (co-author). hiPSCs were obtained from Kyoto University.
Authentication	All cell lines we used have already been used in several groups, and detailes are well described in the literatures.
Mycoplasma contamination	We routinely checked mycloplasma contamination by a PCR kit (Takara).
Commonly misidentified lines (See ICLAC register)	*Name any commonly misidentified cell lines used in the study and provide a rationale for their use.*

Animals and other organisms

Policy information about studies involving animals; ARRIVE guidelines recommended for reporting animal research

Laboratory animals	We used NOD/Scid mice (NIhon Clea) and NSG-mdx-4cv mice (Dr. Kyba at Minnesota U)(5-month old male mice). SPF condition.
Wild animals	We did not use wild mice.
Field-collected samples	N/A
Ethics oversight	All experimental procedures using mice were approved by the Ethical Committees of the National Institute of Neuroscience, National Center of Neurology and Psychiatry (NCNP), Japan, and performed according to the guidelines.

Note that full information on the approval of the study protocol must also be provided in the manuscript.

Flow Cytometry

Plots

Confirm that:

☒ The axis labels state the marker and fluorochrome used (e.g. CD4-FITC).

☒ The axis scales are clearly visible. Include numbers along axes only for bottom left plot of group (a 'group' is an analysis of identical markers).

☒ All plots are contour plots with outliers or pseudocolor plots.

☒ A numerical value for number of cells or percentage (with statistics) is provided.

Methodology

Sample preparation	We treated cells with 0.05% trypsin-1%EDTA and collected by cell scraper. Cells were resuspended in 2%FBS/PBS and incubated with antibodies (1:200 dilution).
Instrument	We used BD FACSAriaFusion.
Software	We used BD FACS Diva and FlowJo(BD).
Cell population abundance	We show cell abundance and percentage in the text.
Gating strategy	We present our gating strategy in the supplementary data.

☒ Tick this box to confirm that a figure exemplifying the gating strategy is provided in the Supplementary Information.
